# A Rare Case of Pancreatic Tail Hydatid Cyst with Incidental Adenocarcinoma of the Pancreatic Body

**DOI:** 10.7759/cureus.3927

**Published:** 2019-01-21

**Authors:** Elbrus Zarbaliyev, Payam Hacısalihoğlu, Dauren Sarsenov

**Affiliations:** 1 General Surgery, Istanbul Yeni Yuzyıl University, Gaziosmanpasa Hospital, Istanbul, TUR; 2 Pathology, Istanbul Yeni Yuzyıl University, Gaziosmanpasa Hospital, Istanbul, TUR; 3 General Surgery, Altunizade Acibadem Hospital, Istanbul, TUR

**Keywords:** pancreatic cyst hydatid, pancreatic cystic lesion, pancreatic cancer, pancreatic echinococcosis

## Abstract

Pancreatic hydatid cyst is a rare disease found mostly in endemic regions. Having no specific clinical signs, it may present with tension related abdominal pain, dyspepsia, a palpable mass, and signs of external pressure on the surrounding organs in accordance with localization of the lesion. Pancreatic carcinoma as a neoplastic pathology with poor prognosis can have various clinical presentations changing with localization of the tumor which sometimes has cystic components. Due to the distinct nature of these pathologies, surgical approach can be fairly different. In this report, we present a case of a 70-year-old patient who had an isolated hydatid cyst in the tail of the pancreas with an incidental pancreatic carcinoma in the corpus of the pancreas. The patient was treated with a subtotal pancreatectomy, having no problems in the postoperative period leading to uncomplicated discharge.

## Introduction

As in other mammalian animals, a human is the end host for Echinococcus granulosus which forms cysts in various anatomical locations. Parasite ova are transported from the small bowel via the hematogenous route; in more than 50% of cases, it affects the liver; it is also able to spread into other organs like the lungs, spleen, brain, bones, retroperitoneal space, and muscles [1-2]. A hydatid cyst is rather rarely seen solely in the pancreas, as in other rare locations it mostly manifests in the pancreas as part of a multiple site involvement [2-3]. Isolated involvement of the pancreas is rare and is mostly seen in endemic regions accounting for nearly 0.2%-1% of the cases [2-4].

On the other hand, pancreatic carcinomas, being very aggressive tumors, are mostly seen in the elderly (45-80 years of age). Tumors localized in the pancreatic body and tail are mostly found incidentally or at advanced stages [5]. In general, the radical treatment of resectable tumors is surgical resection. In advanced stages, surgery (by-pass procedures for obstructive complications) or chemotherapy are reserved for palliation.

In this case, we aim to present a combination of a pancreatic hydatid cyst with an incidental pancreatic carcinoma which was managed surgically.

## Case presentation

**Figure 1:**
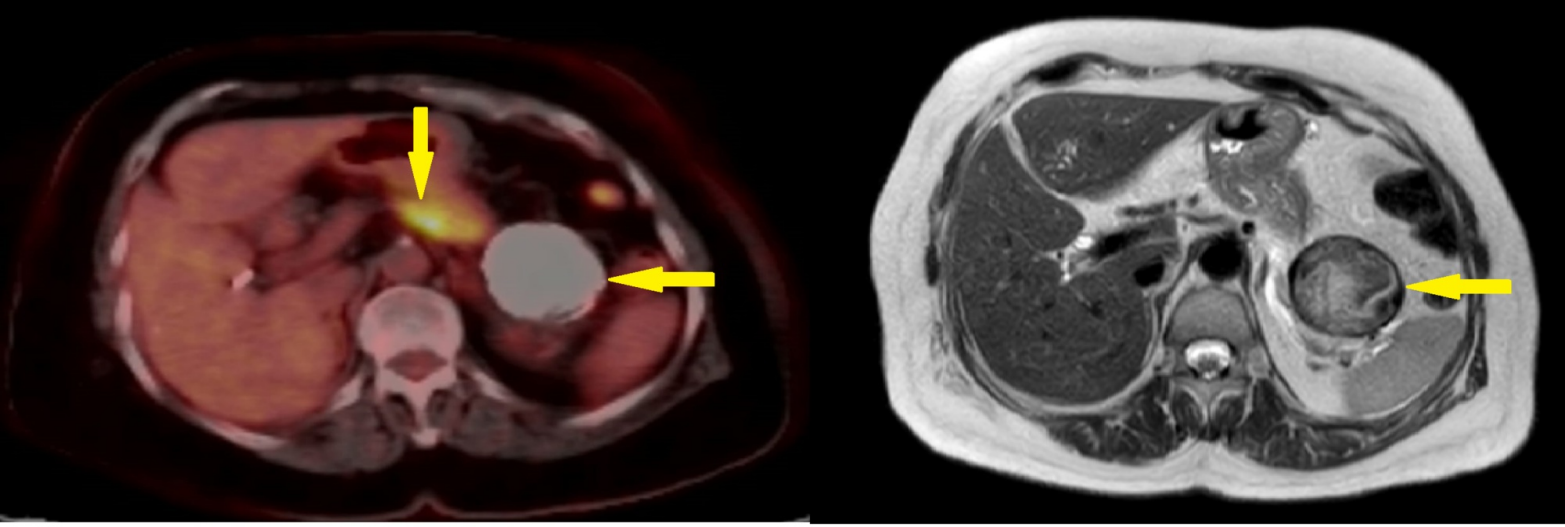
PET-CT fusion and MR images demonstrating pancreatic lesions Solid and cystic lesions in the body and tail of pancreas (arrows). PET-CT: positron emission tomography-computed tomography; MR: magnetic resonance.

A 70-year-old female patient was admitted with epigastric pain and bloating. Abdominal ultrasonography revealed a 6 x 5 cm sized, well-confined cystic lesion without a solid component in the pancreatic tail. There was no pancreatitis history in her anamnesis. The patient was prescribed an upper abdomen magnetic resonance imaging (MRI) scan which showed a cystic lesion with calcified walls in the pancreatic tail along with a 6 x 3 cm hypointense corpus lesion which was invading the splenic vein, at the same time it was showing less contrast uptake when compared to normal pancreatic tissue (Figure 1).

**Figure 2:**
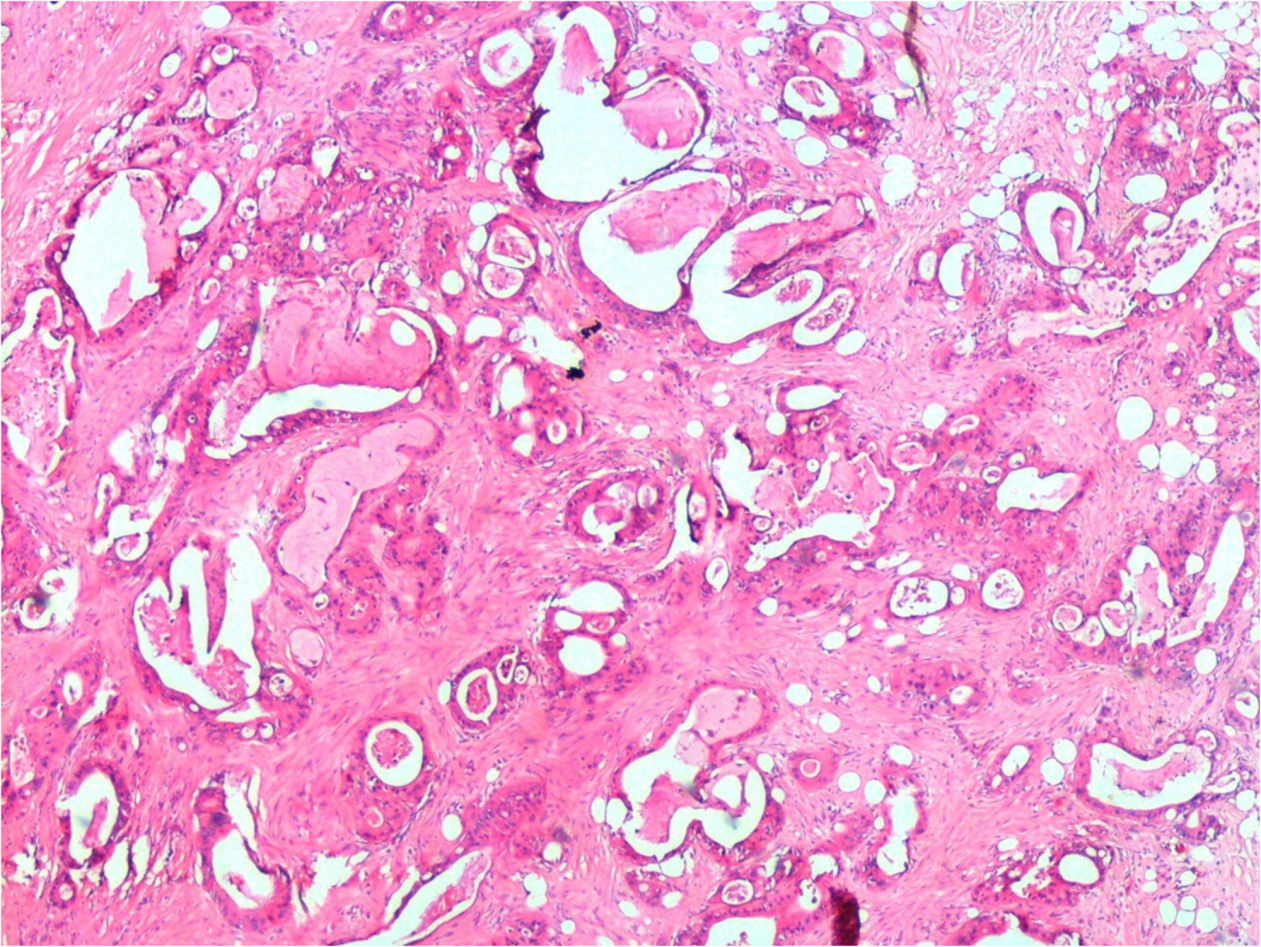
Pancreas adenocarcinoma Moderately differentiated ductal adenocarcinoma (hematoxylin and eosin stain, 40x).

CA 19-9 value was elevated at 1012 IU/ml. The positron emission tomography-computed tomography (PET-CT) scan showed a focally increased fluorodeoxyglucose (FDG) metabolization in the pancreas body with maximum standardized uptake value (SUVmax) of 11.8 without the involvement of the cystic lesion localized in the pancreatic tail. After meticulous evaluation of the tail lesion, it was concluded to be a Type V hydatid cyst without the opportunity of ruling out cystic pancreatic lesions. After the necessary preoperative assessment, the patient was operated on and had a subtotal pancreatectomy with a splenectomy via the left subcostal incision. The pathology report stated that the solid mass was an intermediate grade ductal pancreatic adenocarcinoma with clear surgical margin (Figure 2).

**Figure 3:**
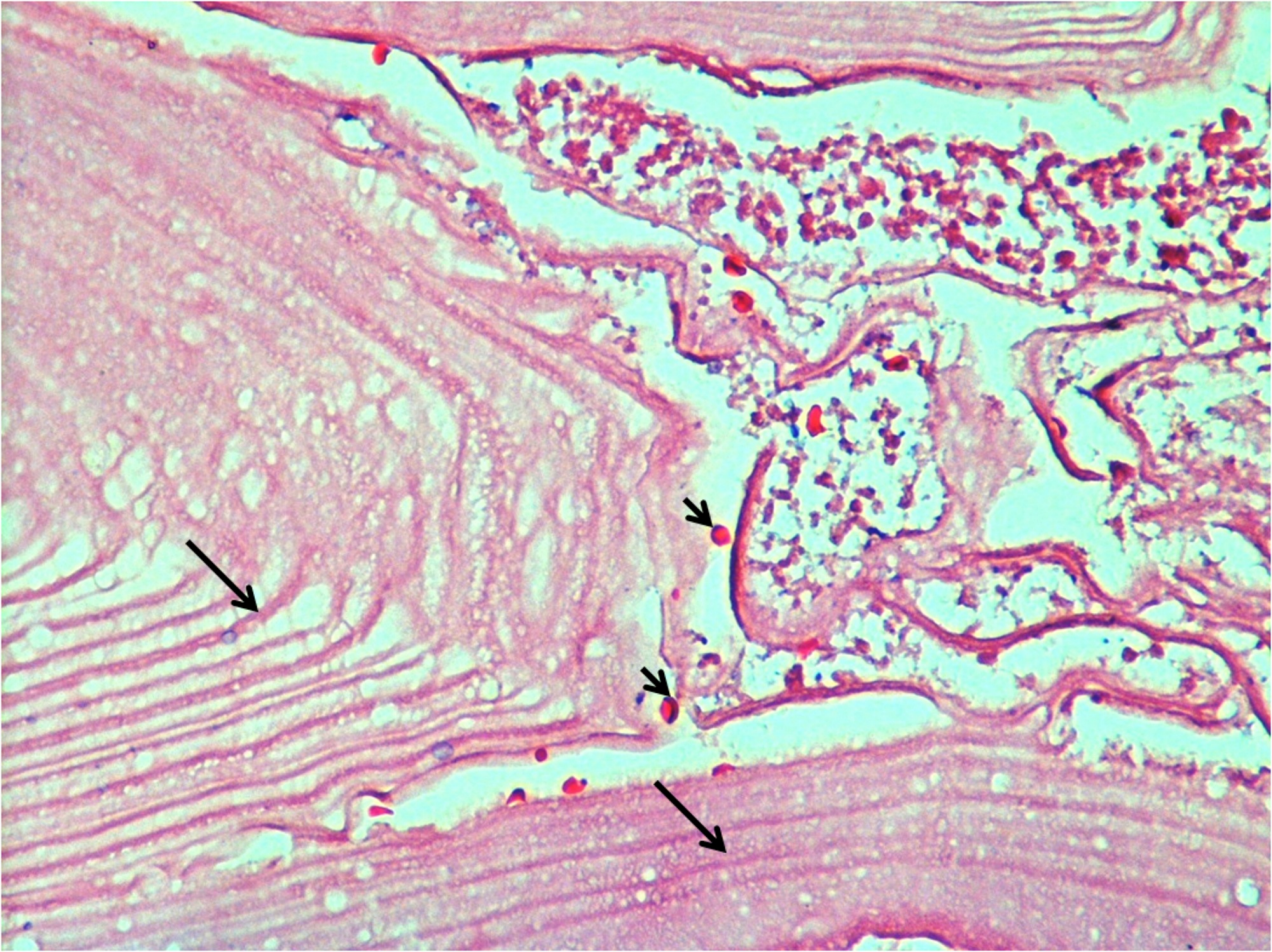
Cyst hydatid The cyst wall has a laminated external layer and a thin germinal layer (arrows) with protoscoleces between layers (arrowheads).

**Figure 4:**
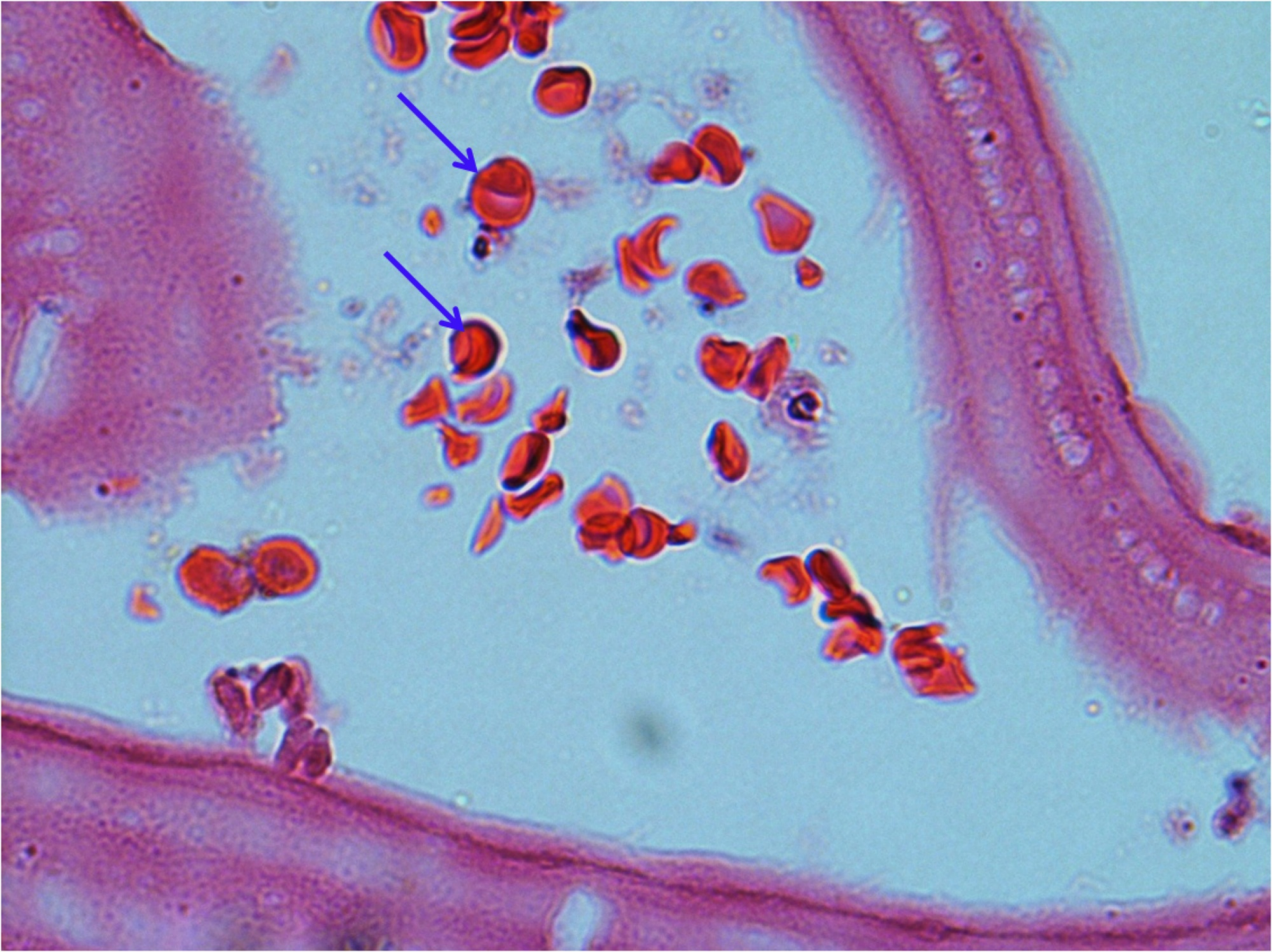
Cyst hydatid protoscoleces Echinococcus granulosus protoscoleces located along the germinal layer seen under higher magnification (arrows).

The cystic lesion was interpreted as an Echinococcus granulosus cyst with all the pathological features present like germinal layer and protoscoleces (Figures 3-4).

The distance between the cyst wall and carcinoma's lateral border was 19 mm without any histological evidence of any relationship between the lesions. After appropriate recovery, the patient was discharged on postoperative day 11. The patient was referred to medical oncology.

## Discussion

Cyst hydatid still manages to stay most frequent zoonotic infection in endemic regions. It starts with Echinococcus Granulosus ova entering gastrointestinal system leading to various clinical presentations. Despite the liver being the first organ to be infested (due to its localization on the way of portal venous flow), the lungs, brain, bones, retroperitoneum, ovaries, and heart can be involved [1,4,6]. The disease can affect multiple organs at once.

Pancreas being a rare location for this disease is not frequently affected even in endemic regions with less than 1% incidence [3]. Isolated pancreas involvement being rare demonstrates distinct features. Head of the pancreas is the most common site of involvement with 57% along with 24% and 19% for corpus and tail respectively [2]. Similar to pancreatic cancer localized in corpus and tail, echinococcal cysts having same localization do manifest with less symptoms and frequency which makes detection of corpus and tail cysts quite incidental. In its turn, pancreatic head lesions can give early symptoms like pain and obstructive jaundice [3]. In our case, at admission, the main symptoms were dyspepsia with epigastric bloating.

Due to being uncommon, hydatid disease of the pancreas can be confused with other cystic lesions of the pancreas. Pancreatic pseudocyst, pancreatic abscess, and cystic neoplasms of the pancreas should be considered in differential diagnosis. The enzyme-linked immunosorbent assay (ELISA) and different imaging methods are used together with clinical suspicion in order to make the differential diagnosis. It should not be forgotten that the ELISA test shows false negativity in 15%-20% of cases [6,7]. In the present case, ELISA for Echinococcus granulosus was negative.

Despite having ultrasonography, abdominal tomography, and MRI as diagnostic modalities, the definitive diagnosis sometimes is made after surgery [2]. Although there is no use of PET-CT for diagnostic purposes in this patient group, some studies have been reported (especially for alveolar hydatid cyst) [8]. In our case, the reason for performing PET-CT was high level of blood CA19-9.

There are therapeutic options such as medical (albendazol 10-15 mg/kg/day), ultrasound-guided percutaneous puncture-aspiration-injection-reaspiration therapy (PAIR), and surgery [9-11]. Although medical treatment provides up to 60%-90% success rate, it also takes a long time while PAIR and surgery combined provide shorter hospital stay and lower morbidity and mortality (6.3-15.8% and 0.0-1.1% respectively) in the treatment of cyst hydatid disease [9,12]. The same approach is preferred in pancreatic hydatid cysts.

Differential diagnosis of hydatid cyst can be made both radiologically and biochemically [9,12]. But due to the heterogeneous nature of the lesion and negative serology tests, there was virtually no possibility to safely rule out true pancreatic cyst with solid and degenerate components in its wall.

Surgical intervention for tail cysts of the pancreas in most cases is reserved for distal pancreatectomy. As a result of the incidental presence of a malignant lesion in our case and no opportunity to safely rule out true cystic lesion of the pancreas, subtotal pancreatectomy was considered appropriate.

To the best of our knowledge, a case of pancreatic cyst hydatid accompanied by pancreatic cancer has not been previously reported due to the rare endemic incidence of pancreatic hydatid disease. Interestingly, it was shown that the course of both diseases is largely influenced by T cell immunity [13-14]. It is thought that hydatid and malignant tumors (especially liver metastases) interact with the organ’s specific local immune systems. Some studies have been done to investigate this idea [15]. In our case, these two pathologies seem to be totally independent.

## Conclusions

Isolated hydatid cysts of the pancreas are rare and may be difficult to distinguish from other cystic lesions of the pancreas. Accordingly, hydatid cysts should be kept in mind for cystic lesions of the pancreas especially for the patients who live in the endemic regions. Further reports should provide more information on this rare combination of adenocarcinoma and hydatid cyst.
